# Acknowledging excellent reviewers and associate editors

**DOI:** 10.1016/j.wroa.2020.100046

**Published:** 2020-02-14

**Authors:** Eberhard Morgenroth

**Affiliations:** a ETH Zürich, Institute of Environmental Engineering, 8093, Zürich, Switzerland; b Eawag, Swiss Federal Institute of Aquatic Science and Technology, 8600, Dübendorf, Switzerland

Today, anybody can upload research reports and data to the internet making research results instantaneously available to the world. In this situation, what is the benefit publishing in a scientific journal such as *Water Research*? In the end, it comes down to trust. The trust of readers in the significance and quality of articles published in *Water Research*. And the trust of authors in the rigor and the fairness of the review process.

How are decisions made at *Water Research*? There is an initial screening by the handling editor to evaluate scope and suitability of the manuscript. At this stage, also manuscripts that do not meet basic quality standards of writing and presentation are rejected (van Loosdrecht and Henze, 2012). Manuscripts that pass the initial screening are sent out to experts in the field for peer review. Once sufficient reviews have been received, the editor takes the ultimate decision to accept, ask for revisions, or reject the manuscript.

There are some concerns and the reputation of peer review has been tainted as the process involves human judgement and there can be mistakes. We, the editors of *Water Research,* are confident that peer review overall works and it provides the necessary basis for editorial decisions that are trusted by readers and authors of the journal. High quality and quick reviews are the foundation of the peer review system.

With an ever increasing number of manuscripts submitted to scientific journals, high quality and rapid peer review may very well be the Achilles heel of scientific publishing. The number of papers submitted to *Water Research* has increased over the past 10 years from 2732 (in 2009) to 5897 (in 2019). We, the editorial board of *Water Research,* want to express our sincere gratitude to *all* the reviewers that help us evaluate these manuscripts submitted to *Water Research*. You support and dedication to *Water Research* is much appreciated. The journal could not exist without your support. Some reviewers and associate editors go beyond the call of duty and they are outstanding in terms of number and in terms of quality of reviews. We dedicate this editorial especially to those reviewers and associated that were truly outstanding in their service in the past two years. Thanks to all reviewers – and especially to our outstanding reviewers and associate editors!

## Outstanding reviewers

**James R. Bolton**, Bolton Photosciences Inc., Edmonton, Alberta, Canada.

**WenHai Chu**, Tongji University, Shanghai, China.

**Dionysios Dionysiou**, University of Cincinnati, Cincinnati, Ohio, United States.

**Xiaohong Guan**, Tongji University, Shanghai, China.

**Jeremy S. Guest**, University of Illinois at Urbana-Champaign, Urbana, Illinois, United States.

**Albert I. Guisasola**, Autonomous University of Barcelona, Barcelona, Spain.

**Zhen (Jason) He**, Washington University in St. Louis, St. Louis, Missouri, United States.

**Peiying Hong**, King Abdullah University of Science and Technology, Thuwal, Kingdom of Saudi Arabia.

**Jin Hur**, Sejong University, Seoul, Korea.

**Simon Judd**, Cranfield University, UK.

**Yunho Lee**, Gwangju Institute of Science and Technology, Korea.

**Heng Liang**, Harbin Institute of Technology, Harbin, China.

**Pascal Saikaly**, King Abdullah University of Science and Technology, Thuwal, Kingdom of Saudi Arabia.

**Paola Verlicchi**, University of Ferrara, Ferrara, Italy.

**Davide Vione**, University of Torino, Torino, Italy.

**Jannis Wenk**, University of Bath, Bath, UK.

## Outstanding associate editors

**Andy Baker**, University of New South Wales, Sydney, New South Wales, Australia.

**Damien Batstone**, University of Queensland, St.Lucia, Queensland, Australia.

**Treavor H. Boyer**, Arizona State University, Tempe, Arizona, United States.

**Rob Eldridge**, (retired), Australia.

**Dionisis Mantzavinos**, University of Patras, Patras, Greece.

**Adrian Oehmen**, New University of Lisbon, Caparica, Portugal.

**Vayalam Venugopalan**, Bhabha Atomic Research Centre, Kalpakkam, India.

## References

[bib1] van Loosdrecht M.C.M., Henze M. (2012). Up-front rejections or which type of paper should I not submit to Water Research. Water Res..

